# Autism traits and mental well-being: the mediating role of social camouflaging and the moderating role of social exclusion and public stigma

**DOI:** 10.1038/s41598-025-20569-7

**Published:** 2025-10-21

**Authors:** Ismail Seçer, Fatmanur Çimen, Sümeyye Ulaş, Eda Tatlı, Feyzanur Saatçı, Abdurrahman Pakiş

**Affiliations:** 1 Department of Psychological Counseling and Guidance, Atatürk University, Erzurum, Turkey; 2 Department of Psychology, Erzurum Technical University, Erzurum, Turkey; 3 Department of Child Development, Van Yüzüncü Yıl University, Van, Turkey; 4 Promotion of Health and Innovation for Well-Being (PHI-WELL), Department of Social Studies, University of Stavanger, Stavanger, Norway; 5 Promotion of Health and Innovation (PHI) Lab, International Network for Well-Being, Linköping, Sweden

**Keywords:** Autism traits, Camouflaging, Mental Well-Being, Social exclusion, Perceived public stigma, Psychology, Risk factors

## Abstract

There is a strong relationship between autism and mental health problems. Autistic individuals are more frequently exposed to stigma and social exclusion in social life, which may lead them to engage in socially desirable behaviors to camouflage themselves. Within this framework, the present study aimed to examine the relationship between autism traits and mental well-being, focusing on social camouflaging behaviors in the context of social exclusion and perceived stigma. The study sample consisted of 548 adults across Turkey, including 432 women (78.8%) and 116 men (21.2%). To test the proposed model, Model 4 and Model 21 developed by Hayes were used. Data were analyzed using SPSS 21 and PROCESS Macro. The findings indicated a negative and significant relationship between autism traits and mental well-being, with social camouflaging mediating this relationship. In addition, the relationship between autism traits and social camouflaging was found to be influenced by social exclusion, while the relationship between social camouflaging and mental well-being was moderated by perceived public stigma. The findings are expected to contribute to broadening perspectives in autism research.

**Supplementary Information:**

The online version contains supplementary material available at 10.1038/s41598-025-20569-7.

## Introduction

Autism Spectrum Disorder (ASD), characterized by impairments in social interaction and communication, along with restricted interests and repetitive behaviors, is an increasingly prevalent neurodevelopmental condition^1,2^. In DSM-IV, Asperger’s Syndrome and Kanner’s Autism were classified as distinct disorders, but in DSM-5, they were merged into a single spectrum, reflecting different severity levels of the same condition^3^. As a result, many cases previously diagnosed as Asperger’s Syndrome are now classified under ASD according to DSM-5. Moreover, the overlap between diagnostic criteria for Social Communication Disorder (SCD) and ASD has emerged as a challenge for clinicians in the diagnostic process^4^. Another diagnostic challenge in ASD is the presence of subthreshold ASD, referring to individuals who exhibit autistic characteristics but do not fully meet the diagnostic criteria for ASD. These individuals display symptoms that significantly resemble those of children diagnosed with ASD, but their social-cognitive and emotional symptoms tend to be milder compared to full ASD. Research indicates that children with subthreshold ASD frequently experience limited friendships, low personal and social adaptation skills, and various emotional problems^1,5,6^. These characteristics suggest that autism or autistic traits may contribute to mental health issues, particularly due to the lack of clear symptom recognition and early diagnosis^1,7^. Since underlying ASD symptoms are not adequately identified and addressed at an early stage, they often remain misunderstood, leading to increased treatment resistance and chronicity^1,8,9^. Findings from the literature highlight a strong relationship between ASD and mental health problems, indicating that individuals with ASD and autism traits exhibit high comorbidity with various psychiatric disorders^10–12^.

Having autism traits may negatively impact mental well-being. Autistic individuals often perceive themselves as having low social support^13^ and frequently experience psychological adjustment issues such as depression and anxiety^6,9,12,14–19^. However, some studies suggest that autistic individuals who feel a sense of belonging to a social group experience fewer mental health problems^19^.

One of the most significant challenges awaiting autistic individuals in social life is the expectation of social exclusion^20^. Social exclusion is defined as “the systematic disadvantaging of certain individuals or groups due to discrimination based on ethnicity, race, religion, gender, sexual orientation, origin, age, disability, migration status, place of residence, etc.”^21^. In other words, it can be understood as “individuals being ignored by others despite their physical presence”^22^. In this context, autistic individuals may strongly perceive the risk of social exclusion due to the challenges they face in social communication and interaction. Social exclusion has been reported to have negative relationships with variables such as functionality, family belonging, self-integrity, subjective well-being, depression, and psychological resilience^23–25^. Therefore, social exclusion is considered a significant threat to the mental health and adaptation processes of autistic individuals^26^.

Another significant issue for autistic individuals is social stigma. Social exclusion, defined as the inability of individuals to integrate into society^27^, particularly affects disadvantaged groups such as people with disabilities^28^. Studies conducted with various populations, including prisoners, older adults^29,30^, university students studying in different cities^27^, and refugees, demonstrate that individuals who experience stigma encounter difficulties in socialization and various psychosocial challenges, which negatively affect their psychological well-being^31^. Furthermore, research indicates that families of individuals with autism are stigmatized by society; this stigmatization significantly undermines their quality of life and leads to stress^32^.

Due to visible autistic behaviors, the fear of exclusion and discrimination is among the intense emotions experienced by autistic individuals^33^. Research indicates that autistic individuals who feel stigmatized tend to perceive greater difficulties in social functioning^34^, lower self-efficacy^35^, and reduced quality of life compared to others^36^. Similarly, individuals who perceive themselves as stigmatized are more likely to adopt dysfunctional coping strategies^35^, experience more severe depressive symptoms, and have higher perceptions of rejection and discrimination^36,37^. In a study conducted with families of individuals with autism, it was reported that negative attitudes and behaviors of society toward individuals with autism and their families place them in a challenging situation, both in terms of facilitating the socialization and integration of individuals with autism into society and regarding the psychological experiences of their families^38^.

Autistic individuals or those with autism traits who experience rejection, stigma, and discrimination may attempt to cope with the resulting stress by adopting socially desirable behaviors. They may observe their surroundings, internalize popular behaviors and social norms, and conceal their true selves in an effort to socialize through this adaptation process^39–41^. The primary motivation behind social camouflaging in autistic individuals is their desire to cope with the threat of social exclusion and rejection while maintaining social adaptation and connection^40^. Autistic individuals often believe they must alter their autism traits to be accepted by society and strive for acceptance by mimicking socially desirable behaviors through camouflaging^40,42^. In this process, they may focus on observing others, internalizing social norms and behavioral patterns, and modeling them to fit into social environments^40,42^. Among the camouflaging behaviors commonly exhibited by autistic individuals are strategies such as *“trying to maintain eye contact*,* using an engaged facial expression*,* asking questions to encourage conversation about the other person*,* and focusing more on listening rather than speaking”*^40^. These strategies may be displayed in simple or complex forms^43^. Contrary to expectations, camouflaging behaviors used by autistic individuals to adapt to social life may lead to negative outcomes. Research has reported strong relationships between camouflaging behaviors and *social anxiety*,* depression*, and *generalized anxiety*, indicating that camouflaging is actually a risk factor^40,44^. Additionally, studies have highlighted that individuals who engage in camouflaging experience higher levels of depressive symptoms and lower feelings of acceptance^45^. In recent years, researchers have increasingly focused on camouflaging behaviors as a coping mechanism used by autistic individuals or those with autism traits to conceal their differences and enhance social acceptance. Although existing research in the literature has contributed to understanding the social behaviors of autistic individuals, it does not yet appear to be at a level that fully explains the nature of camouflaging behaviors. Therefore, there is a need to examine the relationship patterns between camouflaging behaviors, mental health issues, and the triggering factors of exclusion and stigma.

## The current study

A review of the literature reveals that no study has comprehensively examined the relationship between autism traits and mental well-being by considering social exclusion and stigma in individuals who attempt to tolerate this relationship through social camouflaging. In this regard, understanding the relationship processes between mental well-being, social exclusion, and stigma in autistic individuals, as well as identifying the potential mediating role of social camouflaging, is expected to broaden the perspectives of mental health professionals and researchers. Furthermore, it may contribute to a better understanding of the complex nature of autistic individuals’ social behaviors. Therefore, the aim of this study is to determine the moderating roles of social exclusion and stigma and the mediating role of social camouflaging in the relationship between autism traits and mental well-being. In line with this objective, the study seeks to answer the following research questions.

Research question 1: Is there a significant and predictive relationship between autism traits and mental well-being, and what is the mediating role of social camouflaging in this relationship?

Research question 2: What is the moderating role of social exclusion and perceived public stigma in the relationship between autism traits and mental well-being, which is mediated by social camouflaging?

**Fig. 1:**
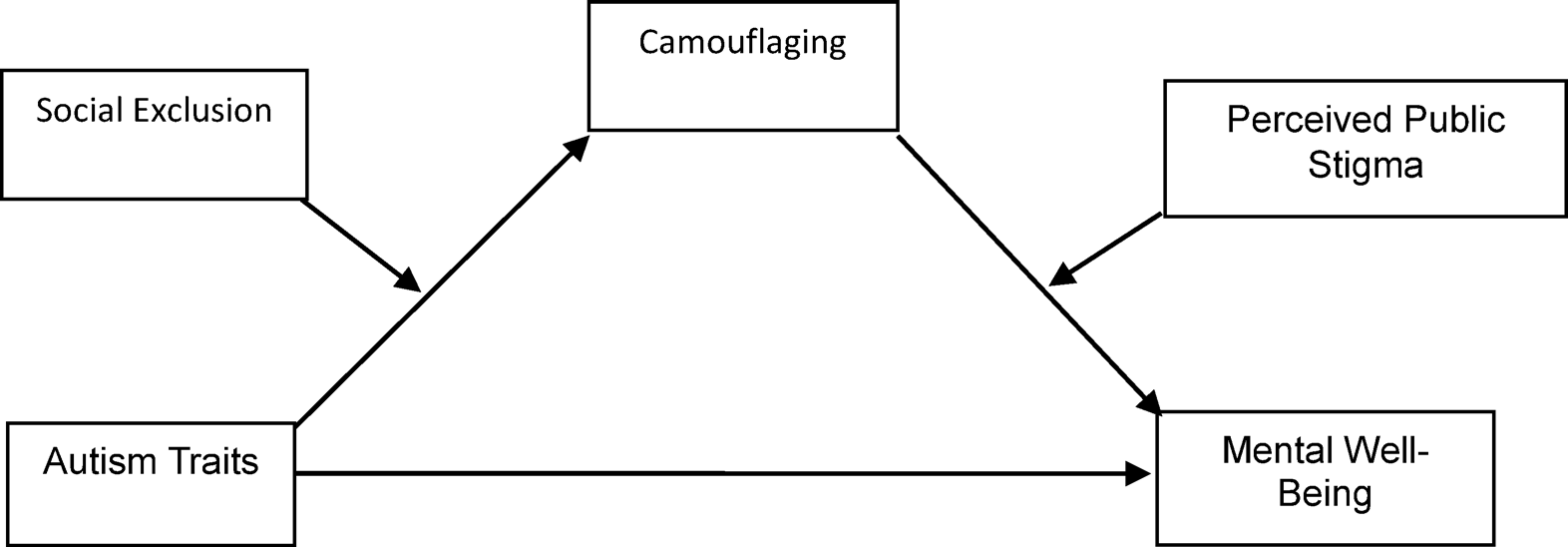
The constructed model.

The model developed in the context of these research questions is presented in Fig. 1.

## Method

### Research sample

The sample of this study consists of 548 individuals from the general population, including 432 women (78.8%) and 116 men (21.2%), with a mean age of 24.67 (SD = 8.07). This number is consistent with the recommended minimum sample size of 300–500 for reliable estimates and also meets the suggested minimum for Structural Equation Modeling (SEM)^46^. Data were collected using the snowball sampling method, and participants were reached through Google Forms. A brief introduction explaining the purpose of the study was provided, emphasizing voluntary participation. Participants who agreed to take part were directed to an online survey form. In addition to demographic information, participants completed the scales selected for this study. Each question in the survey was mandatory, ensuring that no missing data were present. Demographic information regarding the study sample is presented in Table 1.

**Table 1:** Demographic data.

		*N* (%)
Gender	Female	432 (78.8)
	Male	116 (21.2)
Age	Mean (sd)	24.67 (8.07)
Total		548 (100,0)

### Measures

*Comprehensive Autistic Trait Inventory* (CATI): To measure autism traits, the Comprehensive Autistic Trait Inventory (CATI) was developed as a 42-item, 5-point Likert-type scale^47^. The scale consists of six subdimensions: repetitive behaviors, sensory sensitivity, cognitive rigidity, social interaction, communication, and social camouflaging. Maintaining the original structure, a short version of the scale with 24 items was developed^48^. In this study, the Turkish adaptation of the short version was used^49^. As an indicator of autism traits, five subdimensions of the scale were utilized: repetitive behaviors, sensory sensitivity, cognitive rigidity, social interaction, and communication. The psychometric structure of the scale was examined through Confirmatory Factor Analysis (CFA), which indicated an acceptable model fit (χ2/df: 2.456, CFI: 0.92, IFI: 0.92, RMSEA: 0.05). Reliability analyses demonstrated that the scale had high reliability (α = 0.81).

*Social Camouflaging*: In this study, social camouflaging was measured using the 4-item social camouflaging subscale of the CATI-SF scale. The psychometric structure of the scale was examined through Confirmatory Factor Analysis (CFA), which indicated a good model fit (χ2/df: 3.044, CFI: 97, IFI: 97, RMSEA: 0.06).

*Perceived Public Stigma* (PPS): Perceived public stigma was measured using an eight-item scale, adapted from a previous study^50,51^. The scale is unidimensional, with items rated on a six-point Likert scale, where higher scores indicate greater perceived public stigma. The Turkish adaptation of the scale was conducted^52^. Confirmatory Factor Analysis (CFA) demonstrated good model fit (χ2/df = 2.67, CFI = 0.98, IFI = 0.98, RMSEA = 0.55), and the Cronbach’s alpha internal consistency coefficient was found to be high (α = 0.76), confirming the scale’s validity and reliability for use in the Turkish cultural context.

*Warwick-Edinburgh Mental Wellbeing Scale* (WEMWBS): The scale developed to measure individuals’ mental well-being was used in this study^53,54^. The scale consists of 14 items, rated on a five-point Likert scale, and is unidimensional. The internal consistency reliability of the scale was reported with a Cronbach’s alpha coefficient of 0.89, and the test-retest reliability correlation coefficient was 0.83, confirming the scale’s validity and reliability for use in the Turkish cultural context.

*Ostracism Short Scale* (OSS): Individuals’ experiences of social exclusion were measured using the OSS, developed in an earlier study^55^. The scale consists of four items, rated on a seven-point Likert scale, and is unidimensional. Higher scores indicate higher levels of exclusion experiences within the past two months. The Turkish adaptation of the scale was conducted^56^. Confirmatory Factor Analysis (CFA) demonstrated good model fit (χ2/df = 1.87, CFI = 0.99, IFI = 0.99, RMSEA = 0.04), and the Cronbach’s alpha reliability coefficient was found to be high (α = 0.90), confirming the scale’s validity and reliability for use in the Turkish cultural context.

### Data analysis

To evaluate the research hypotheses formulated in line with the study’s objective, a mediated moderation model was constructed. Mediated moderation occurs when the moderating effect between two variables is mediated by another variable^57^. For data analysis and model testing, SPSS 21 version and Macro 4.3 programs were used. In this context, first, a mediation model was developed, and the mediating relationships between the variables were tested. Then, moderator variables were included in the mediation model, and the integrated model was tested. Model 4 was used to test Hypothesis 1 and Hypothesis 2, while PROCESS Model 21 was used to test Hypothesis 2. First, a mediation model was developed to examine the mediated effects among the variables. Next, moderator variables were incorporated to assess moderated effects within the mediation framework, allowing the model to capture both indirect and conditional relationships. To assess the significance of indirect effects, bootstrapping was performed with 5,000 resamples (95% confidence intervals [CI]; Hayes, 2018). To test the research hypotheses, chi-square, Comparative Fit Index (CFI), Tucker-Lewis Index (TLI), Root Mean Square Error of Approximation (RMSEA), and Standardized Root Mean Square Residual (SRMR) values were used. χ2/df < 5, CFI and IFI > 0.90, RMSEA < 0.08, and SRMR < 0.10 are all considered indicators of acceptable model fit^58,59^. In mediation analyses, the criteria proposed in previous research were used as a reference to examine the relationships between the variables^60^.

## Results

### Preliminary analysis

Descriptive statistics and correlations for the research variables are presented in Table 2.

**Table 2:** Descriptive statistics and correlation table.

	Mean (sd)	Skew.	Kurt.	1	2	3	4	5
CATI	54.51 (10.31)	− .537	.390	1	0.529^**^	− 0.109^*^	− 0.045	0.187^**^
SC	11.84 (2.77)	− .074	.338		1	0.032	− 0.044	0.185^**^
WEMWBS	51.47 (11.12)	− .485	.239			1	− 0.004	− 0.361^**^
PPS	21.64 (7.48)	− .041	− .683				1	0.083
OSS	9.72 (5.68)	1.159	.821					1

*CATI: Comprehensive Autistic Trait Inventory, SC: Social Camouflage, WEMWBS: Warwick-Edinburgh Mental Wellbeing Scale, PSS: Perceived Public Stigma, OSS: Ostracism Short Scale, **p < 0.01.

To determine whether normality was met, the skewness and kurtosis values of each variable were examined. The values were found to be within the acceptable range, indicating that the data followed a normal distribution (skewness <|3| and kurtosis <|10|; Kline, 2016). An analysis of correlation values revealed significant positive relationships between autism traits and social camouflaging (*r* = 0.529) and autism traits and social exclusion (*r* = 0.187). In contrast, a significant negative relationship was observed between autism traits and mental well-being (*r* = − 0.109). However, no significant relationship was found between autism traits and perceived public stigma. In addition, a significant positive relationship was found between social camouflaging and social exclusion (*r* = 0.185). No significant relationship was observed between mental well-being and perceived public stigma, whereas a significant negative relationship was found between mental well-being and social exclusion (*r* = − 0.361).

**Fig. 2:**
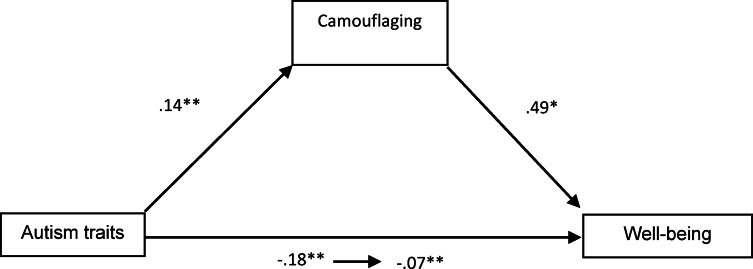
Mediating effect of camouflaging, **p < 0.01, * *p* < 0.05.

Within the scope of the study, to evaluate RQ1, formulated as *“Is there a significant and predictive relationship between autism traits and mental well-being*,* and what is the mediating role of social camouflaging in this relationship?”*, data analyses were conducted using PROCESS Model 4. The findings obtained from the analyses are presented in Fig. 2.

An examination of Fig. 2 reveals that autism traits have a direct and significant positive effect on social camouflaging (β = 0.14, *p* < 0.001) and a direct and significant negative effect on mental well-being (β= − 0.18, *p* < 0.001). In addition, social camouflaging has a direct and significant positive effect on mental well-being (β = 0.49, *p* < 0.005). When social camouflaging was included in the model, the significant effect between autism traits and mental well-being persisted, but this effect decreased substantially compared to the direct effect. Therefore, it can be concluded that social camouflaging plays a significant mediating role in the relationship between autism traits and mental well-being (indirect effect = 0.070, SE = 0.034, *p* < 0.001, %95CI [0.005, 0.141]).

Within the scope of the study, to evaluate RQ2, formulated as *“What is the moderating role of social exclusion and perceived public stigma in the relationship between autism traits and mental well-being*,* which is mediated by social camouflaging?”*, data analyses were conducted using PROCESS Model 21. The findings obtained from the analyses are presented in Table 3.

**Table 3:** The moderating role of social exclusion and perceived public stigma in the relationship between autism traits and mental Well-Being, mediated by social Camouflaging.

	*R* ^2^	F	β	SE	t	*p*	LLCI	ULCI
Model 1	0.295	75.885**						
AT			0.175	0.018	9.399	0.000	0.1385	0.2117
OSS			0.292	0.106	2.737	0.006	0.0825	0.5018
Interaction 1			− 0.004	0.001	− 2.364	0.018	− 0.0081	− 0.0007
Model 2	0.187	4.963**						
AU			− 0.181	0.053	− 3.382	0.000	− 0.2868	− 0.0761
SC			1.751	0.518	3.379	0.000	0.7335	2.770
PPS			0.696	0.276	2.518	0.012	0.1533	1.239
Interaction 2			− 0.058	0.022	− 2.620	0.009	− 0.1023	− 0.0146

*CATI: Comprehensive Autistic Trait Inventory, SC: Social Camouflage, WEMWBS: Warwick-Edinburgh Mental Wellbeing Scale, PSS: Perceived Public Stigma, OSS: Ostracism Short Scale, İnteraction 1: autism traits × Ostracism; Interaction 2: Social Camouflage × Perceived Public Stigma, **p < 0.01, * *p* < 0.05.

An examination of Table 3 reveals that, in Model 1, both autism traits (β = 0.175, *p* < 0.01) and social exclusion (β = 0.292, *p* < 0.01) have a significant effect on social camouflaging. However, the interaction effect between autism traits and social exclusion has a significant negative effect on social camouflaging (β = − 0.004, *p* < 0.05). In Model 2, autism traits (β = − 0.181, *p* < 0.01) have a significant negative effect on mental well-being, while social camouflaging (β = 1.751, *p* < 0.01) and perceived public stigma (β = 0.696, *p* < 0.05) have significant positive effects on mental well-being. However, the interaction effect between social camouflaging and perceived public stigma has a significant negative effect on mental well-being (β = − 0.058, *p* < 0.01). The change in R2 value after adding Interaction 1 was 0.029, and the change in R² value after adding Interaction 2 was 0.018. Therefore, both interaction effects significantly contributed to the model (*p* < 0.05).

**Fig. 3:**
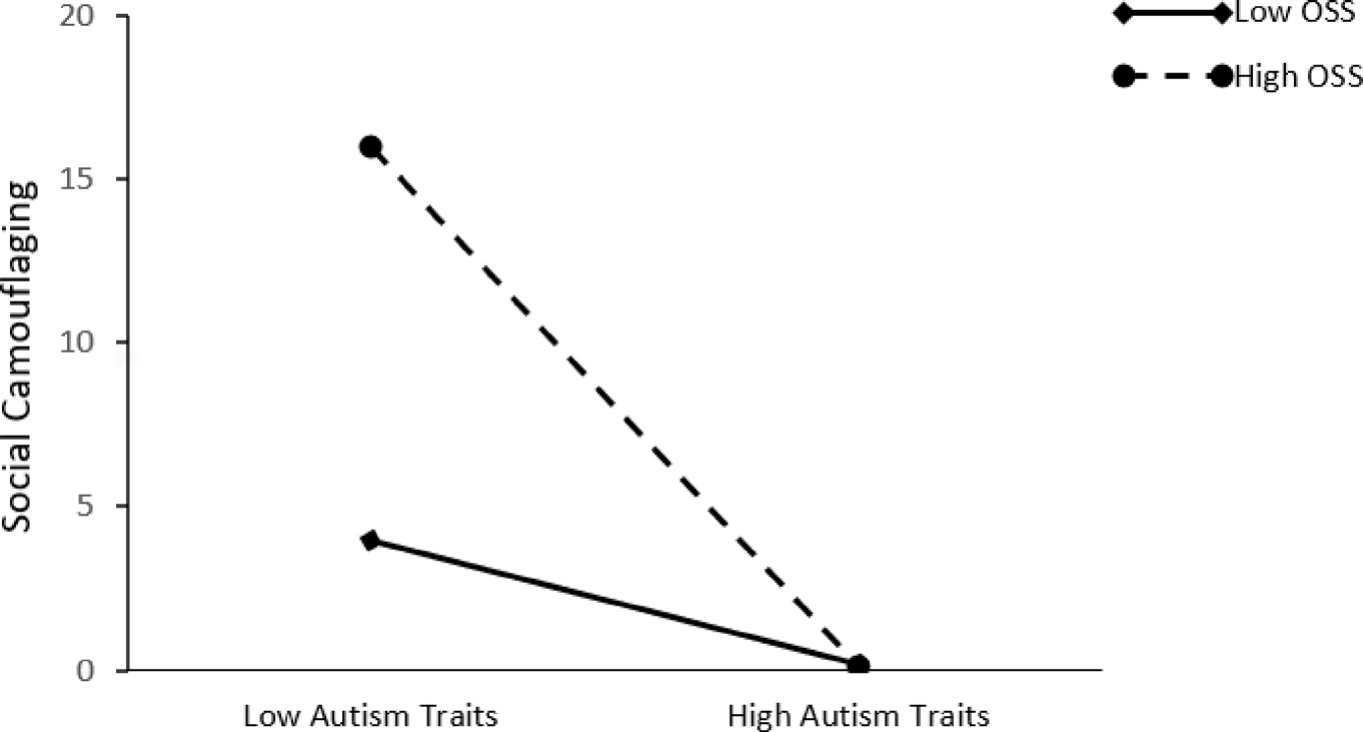
The interaction effect of social exclusion on the relationship between autism traits and social camouflaging.

**Fig. 4:**
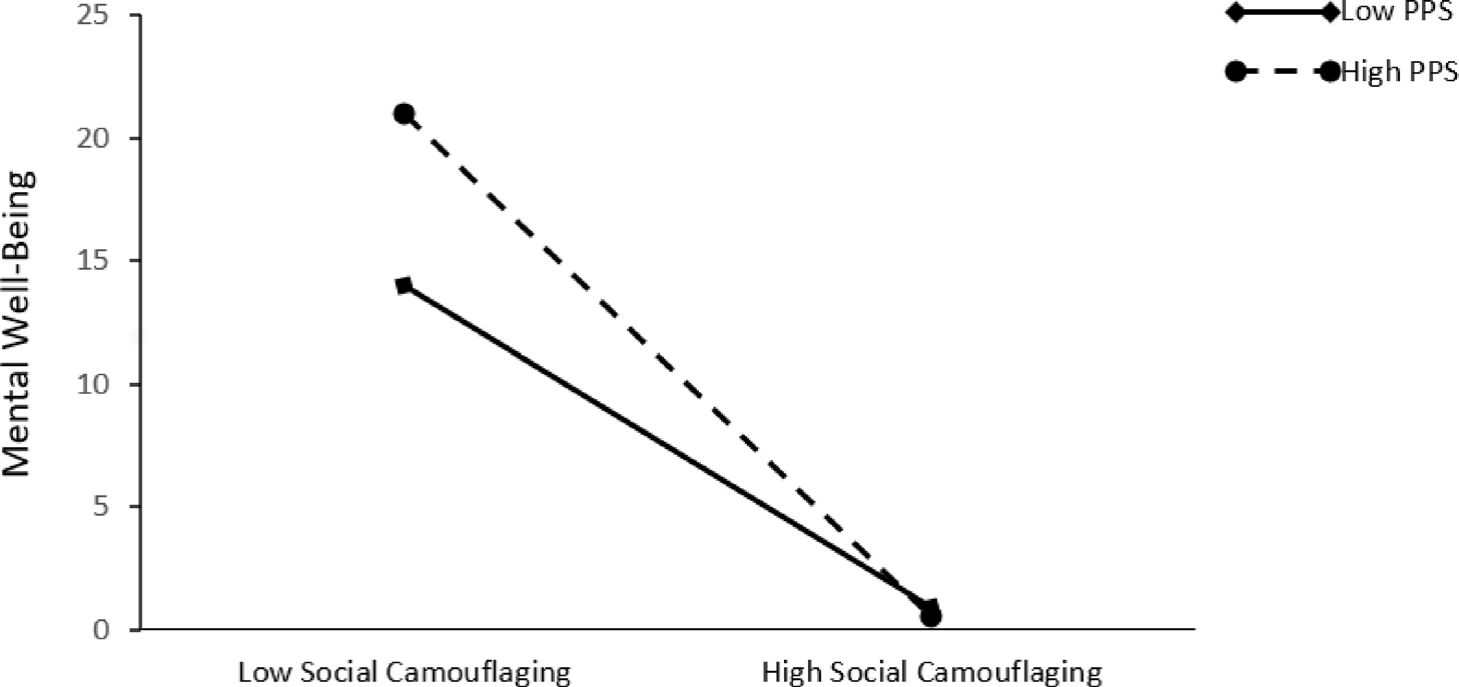
The interaction effect of perceived public stigma on the relationship between social camouflaging and mental well-being.

An examination of Fig. 3 reveals that the increase in social camouflaging is significant at both low and high levels of social exclusion. As the level of social exclusion increases (M + 1SD), a negative slope emerges, indicating that when social exclusion is at its highest level, social camouflaging decreases (simple slope = 0.104, t = 6.080, *p* < 0.01). Thus, it can be concluded that at the highest levels of autism traits, social exclusion directly influences social camouflaging. When social exclusion is low (M − 1SD), the predictive effect of autism traits on social camouflaging decreases (simple slope = 0.157, t = 12.201, *p* < 0.01).

Examining Fig. 4 reveals that the increase in mental well-being is significant at both low and high levels of perceived public stigma. As perceived public stigma increases (M + 1SD), a negative slope emerges, indicating that when perceived public stigma is at its highest level, mental well-being is at its lowest (simple slope = 0.523, t = 2.622, *p* < 0.01). Thus, it can be concluded that when social camouflaging is at its highest level, perceived public stigma directly influences mental well-being. Moreover, when perceived public stigma is low (M − 1SD), the predictive effect of social camouflaging on mental well-being weakens (simple slope = 0.932, t = 3.596, *p* < 0.01).

## Conclusion and discussion

This study focused on the effect of autism traits on mental well-being and identified social camouflaging as a key mechanism explaining the indirect pathway between autism traits and mental well-being. Mediation analyses showed that higher levels of autism traits predicted increased social camouflaging, which in turn was associated with lower mental well-being. Beyond this indirect effect, moderation analyses highlighted that social exclusion conditionally influenced the likelihood of engaging in camouflaging behaviors, while perceived public stigma significantly shaped the strength of the relationship between camouflaging and mental well-being. Taken together, these findings demonstrate a moderated mediation process, in which social camouflaging transmits the effect of autism traits onto mental well-being, while the magnitude of this indirect association varies depending on levels of social exclusion and public stigma. To the authors’ knowledge, this study represents the first comprehensive investigation that simultaneously examines indirect and conditional processes underlying the association between autism traits and mental well-being.

The findings of this study indicate that autism traits significantly and negatively predict mental well-being. This result aligns strongly with the existing literature, which reports that individuals with ASD frequently experience psychological adjustment issues such as depression and anxiety, as well as other mental health difficulties^12,14–19,61^. This result highlights that individuals with ASD or autism traits constitute a high-risk group for mental health issues. It also underscores the necessity of early diagnosis and the implementation of interventions that support social skill development. PCIT interventions were reported to be effective in supporting early adaptation and social skill development in children with subthreshold ASD^62^. Thus, early interventions play a critical role in reducing risks associated with adolescence, youth, and adulthood for these individuals^13,63^.

The second focus of this study is the mediating role of social camouflaging in the relationship between autism traits and mental well-being. The findings revealed that camouflaging behaviors, which autistic individuals adopt to facilitate socialization, play a significant mediating role in this relationship. Although this finding is based on limited literature, it may contribute to a better understanding of mental well-being in autistic individuals^13,64–67^. In a study examining the level of social awareness toward individuals with autism, it was revealed that societal attitudes toward ASD were generally negative^68^. Furthermore, these attitudes were found to be significantly associated with dimensions of social exclusion such as discrimination, stigmatization, and psychological health. In one study, it was revealed that behavior patterns specific to ASD were perceived as “strange” by society and reflected toward individuals with ASD and their families through hurtful words and looks, which in turn restricted their social lives^69^. In line with this, research has shown that individuals with autism often engage in camouflaging behaviors to adapt to their social environment and to avoid stigmatization^13,65,66,70^.

It is suggested that autism traits do not directly impact mental well-being, but rather, the negative effect emerges through social camouflaging. In other words, autistic individuals engage in camouflaging efforts, and the anxiety and stress caused by these efforts exert pressure on their mental well-being. Although paradoxical, autistic individuals face greater mental health challenges in pursuit of social acceptability. It has been argued that autistic individuals consciously use camouflaging, gaining a significant advantage by experiencing less stigma and exclusion^44,71,72^. However, despite these positive outcomes, the strain on mental well-being persists. Thus, the failure of camouflaging efforts to achieve social acceptance, excessive motivation to fit in, and the pressure associated with hiding autism traits may contribute to various mental health issues and negatively impact mental well-being^44,66,72–76^. Recent research supports this claim, reporting that autistic individuals who engage in intense camouflaging frequently experience mental health problems such as anxiety, depression, and burnout^65,77,78^. In light of this, there is a clear need for early interventions aimed at strengthening the social communication and interaction skills of autistic individuals. Due to weak social skills, they often feel excluded and stigmatized, leading them to resort to camouflaging as a means of social acceptance, which places them in a high-risk area. This high-risk area ultimately contributes to greater mental health challenges and lower mental well-being in autistic individuals.

Another significant finding of this study concerns the moderating role of social exclusion in the mediating effect of camouflaging on the relationship between autism traits and mental well-being. The results indicate a positive relationship between autism traits and camouflaging, yet they also highlight that perceived social exclusion is a key determinant in this relationship. Accordingly, autistic individuals who perceive themselves as socially excluded are more likely to engage in camouflaging behaviors. In this context, it is noteworthy that camouflaging behavior is linked to the efforts of autistic individuals to gain social acceptance, yet these processes have negative psychological consequences^79^. Excessive camouflaging has been found to lead to mental health issues such as depression and anxiety^80^. Social exclusion, loneliness, and isolation further exacerbate these challenges, contributing to lower quality of life and increased mental health problems in autistic individuals^81^. Social camouflaging suppresses individuals’ authentic identities, which in the long run deepens feelings of loneliness and isolation^43^. Specifically, an increase in perceived social exclusion heightens individuals’ efforts to adapt to environmental demands, which in turn negatively affects their mental health. Experiences of exclusion and rejection in social environments lead autistic individuals to increase their use of camouflaging strategies, which have negative effects on their mental health^82^. The study emphasized that camouflaging is linked to efforts for social acceptance, yet in the long run, these processes can result in anxiety, depression, and identity confusion. In conclusion, perceived social exclusion drives autistic individuals to engage more frequently in social camouflaging strategies, making it a key factor in exacerbating mental health issues.

Another notable finding of the study concerns the moderating role of stigma in the relationship between camouflaging behavior and mental well-being among autistic individuals. The results indicate that perceived public stigma influences social camouflaging and, consequently, impacts mental well-being in autistic individuals. Accordingly, autistic individuals who perceive high levels of stigma tend to engage in more camouflaging behaviors, which in turn increases the pressure on their mental well-being. Findings from the literature support the established link between stigma and camouflaging^83,84^. Thus, as autistic individuals feel more stigmatized, they become increasingly focused on concealing their autism traits, which may lead to the development of a different persona, placing further strain on their mental well-being^85^. In addition, some perspectives suggest that camouflaging behaviors used to counter stigma function as a form of “survival strategy” in social life for autistic individuals^86^. Although paradoxical, autistic individuals engage in camouflaging to gain social acceptance and enhance well-being, yet they ultimately face a greater risk of stigma and a heightened compromise of their mental well-being.

### Limitations

This study examined the mediating role of social camouflaging in the relationship between autism traits and mental well-being, as well as the moderating effects of social exclusion and perceived public stigma. To achieve this objective, all adults over the age of 18 were included in the study sample without any specific selection criteria. A key limitation of this study is the difficulty of accessing a specific sample of diagnosed autistic individuals, meaning that the research sample could not be categorized into autistic and non-autistic groups. As a result, this study did not examine the impact of having an official autism diagnosis on the relationship between autism traits and mental well-being. The findings of the present study should be interpreted within the framework of autism traits as they manifest in the general population. Future investigations are encouraged to take this factor into account in order to provide a more comprehensive and nuanced understanding of the phenomena under study. Furthermore, as the current research employed a quantitative design to examine the relationships among variables, participants’ subjective experiences were not captured. Accordingly, qualitative or mixed-method approaches focusing on the interplay between autism traits and mental well-being could yield richer insights and make significant contributions to the literature.

## Supplementary Information

Below is the link to the electronic supplementary material.

Supplementary Material 1

## Data Availability

The raw data supporting the conclusions of this article will be made available by the corresponding author, without undue reservation.
