# Supplementary material for: Autism traits and mental well-being: the mediating role of social camouflaging and the moderating role of social exclusion and public stigma
Source: Sci Rep. 2025 Oct 21;15:36633. doi: 10.1038/s41598-025-20569-7 (PMC12540668; doi:10.1038/s41598-025-20569-7)
Supplement: Supplementary file 1 — Supplementary Material 1 [file 41598_2025_20569_MOESM1_ESM.docx]

**Autism Traits and Mental Well-Being: The Mediating Role of Social Camouflaging and the Moderating Role of Social Exclusion and Public Stigma**

**İsmail SEÇER^*^**

University of Ataturk, Erzurum, Turkey

[ismail.secer@atauni.edu.tr](mailto:ismail.secer@atauni.edu.tr)

**Fatmanur ÇİMEN**

University of Ataturk, Erzurum, Turkey

[f.cimen@auauni.edu.tr](mailto:f.cimen@auauni.edu.tr)

**Sümeyye ULAŞ**

Erzurum Technical University, Erzurum, Turkey

[sumeyye.ulas@erzurum.edu.tr](mailto:sumeyye.ulas@erzurum.edu.tr)

**Eda TATLI**

University of Ataturk, Erzurum, Turkey

[edatatli2580@gmail.com](mailto:edatatli2580@gmail.com)

**Feyzanur SAATÇI**

University of Ataturk, Erzurum, Turkey

feyzanur_saatci19@trabzon.edu.tr

**Abdurrahman PAKİŞ**

University of Yüzüncüyıl, Van, Turkey

[kpakish17@gmail.com](mailto:kpakish17@gmail.com)

***Correspondance author**: İsmail SEÇER, University of Ataturk, Erzurum, Turkey

[ismail.secer@atauni.edu.tr](mailto:ismail.secer@atauni.edu.tr)
